# Empagliflozin treatment rescues abnormally reduced Na^+^ currents in ventricular cardiomyocytes from dystrophin-deficient *mdx* mice

**DOI:** 10.1152/ajpheart.00729.2023

**Published:** 2023-12-15

**Authors:** Jakob Sauer, Jessica Marksteiner, Elena Lilliu, Benjamin Hackl, Hannes Todt, Helmut Kubista, Christopher Dostal, Bruno K. Podesser, Attila Kiss, Xaver Koenig, Karlheinz Hilber

**Affiliations:** ^1^Department of Neurophysiology and Pharmacology, Center for Physiology and Pharmacology, Medical University of Vienna, Vienna, Austria; ^2^Center for Biomedical Research and Translational Surgery, Medical University of Vienna, Vienna, Austria

**Keywords:** arrhythmias, cardiomyocyte sodium currents, Duchenne muscular dystrophy, empagliflozin, mdx mice

## Abstract

Cardiac arrhythmias significantly contribute to mortality in Duchenne muscular dystrophy (DMD), a severe muscle illness caused by mutations in the gene encoding for the intracellular protein dystrophin. A major source for arrhythmia vulnerability in patients with DMD is impaired ventricular impulse conduction, which predisposes for ventricular asynchrony, decreased cardiac output, and the development of reentrant circuits. Using the dystrophin-deficient *mdx* mouse model for human DMD, we previously reported that the lack of dystrophin causes a significant loss of peak Na^+^ current (*I*_Na_) in ventricular cardiomyocytes. This finding provided a mechanistic explanation for ventricular conduction defects and concomitant arrhythmias in the dystrophic heart. In the present study, we explored the hypothesis that empagliflozin (EMPA), an inhibitor of sodium/glucose cotransporter 2 in clinical use to treat type II diabetes and nondiabetic heart failure, rescues peak *I*_Na_ loss in dystrophin-deficient ventricular cardiomyocytes. We found that *I*_Na_ of cardiomyocytes derived from *mdx* mice, which had received clinically relevant doses of EMPA for 4 wk, was restored to wild-type level. Moreover, incubation of isolated *mdx* ventricular cardiomyocytes with 1 µM EMPA for 24 h significantly increased their peak *I*_Na_. This effect was independent of Na^+^-H^+^ exchanger 1 inhibition by the drug. Our findings imply that EMPA treatment can rescue abnormally reduced peak *I*_Na_ of dystrophin-deficient ventricular cardiomyocytes. Long-term EMPA administration may diminish arrhythmia vulnerability in patients with DMD.

**NEW & NOTEWORTHY** Dystrophin deficiency in cardiomyocytes leads to abnormally reduced Na^+^ currents. These can be rescued by long-term empagliflozin treatment.

## INTRODUCTION

Cardiac arrhythmias significantly contribute to mortality in Duchenne muscular dystrophy (DMD), a fatal disease caused by dystrophin deficiency (1, 2). Impaired ventricular impulse conduction, predisposing for ventricular asynchrony and the development of reentrant circuits, is a major source of arrhythmias in patients with DMD. Using the dystrophin-deficient *mdx* mouse model for human DMD (3), we and others previously reported that the lack of dystrophin causes a significant peak Na^+^ current (*I*_Na_) loss both in ventricular cardiomyocytes of the working myocardium (4–6) and in Purkinje fibers (7, 8), ventricular myocytes specialized for rapid electrical impulse conduction. These findings provided a mechanistic explanation for ventricular conduction defects and concomitant arrhythmias in the dystrophic heart.

Empagliflozin (EMPA), an inhibitor of sodium/glucose cotransporter 2 (SGLT2) in clinical use to treat type II diabetes and as of late also nondiabetic heart failure (HF) (9, 10), was recently shown to modulate cardiac Na^+^ channels. Thus, application of the drug selectively inhibited the detrimental late *I*_Na_, which occurs in ventricular cardiomyocytes originating from mouse models for HF (11–13). EMPA may thereby exert an antiarrhythmic effect. Dago et al. (14) recently tested the effects of 24-h incubation with EMPA at a therapeutically relevant concentration (1 µM) on *I*_Na_ in cardiomyocytes derived from human-induced pluripotent stem cells. These authors found that EMPA treatment significantly increases peak *I*_Na_, suggesting an upregulation of Na^+^ channel expression under lasting presence of the drug. If EMPA would exert a similar effect in dystrophin-deficient ventricular cardiomyocytes, the drug could be used to counteract peak *I*_Na_ loss in the dystrophic heart.

In this study, we used the *mdx* mouse model for DMD to test the hypothesis that long-term treatment with EMPA rescues peak *I*_Na_ loss in dystrophin-deficient ventricular cardiomyocytes.

## METHODS

### Ethical Approval

The investigation conformed to the guiding principles of the Declaration of Helsinki and coincides with the rules of the Animal Welfare Committee of the Medical University of Vienna. The experimental protocols, which were applied during the study, were approved by the Austrian Science Ministry. The respective ethics vote has the following number: BMWFW-66.009/0175-WF/V/3b/2015.

### Mice and Long-Term EMPA Treatment

Dystrophin-deficient *mdx* mice on the BL10 background (C57BL/10ScSn-Dmdmdx/J) and wild-type control mice (C57BL/10ScSnJ) in an age range between 16 and 23 wk were used for the experiments. These two mouse lines were originally purchased from Charles River Laboratories. Only male mice were used in this study because of the X-linked inheritance of DMD and potential translational relevance to human patients as in Haffner et al. (15). Genotyping of the mice was performed using standard PCR assays. A cohort of *mdx* mice received EMPA (MedChemExpress) via the drinking water. Treatment started at 12 wk of age and lasted for 4 wk. Drinking water containing EMPA was freshly prepared on a weekly basis. First, EMPA was dissolved in dimethyl sulfoxide (DMSO) to obtain a 50 mg/mL stock solution. Then, the stock solution was diluted in drinking water to achieve a final EMPA concentration of 0.1 mg/mL (0.2% DMSO). In consideration of a mean water intake of 5 mL/day and a mean body weight of 33 g, the EMPA concentration of 0.1 mg/mL in the drinking water resulted in an EMPA dose of ∼15 mg/kg body wt/day, a dose lying within the standard range for studies with mice (16–19). Administering EMPA at such a typical dose to mice was previously shown to lead to plasma concentrations comparable with therapeutic EMPA plasma concentrations in humans (19). During treatment with 15 mg/kg/day EMPA, the water intake of the *mdx* mice was twofold increased when compared with that of untreated control *mdx* mice. This confirmed that the applied EMPA dose was effective and led to SGLT2 inhibition. Mice of the control groups, which drank ∼2.5 mL/day, were exposed to drinking water comprising 0.4% of the solvent DMSO for 4 wk.

### Isolation of Ventricular Cardiomyocytes and Drug Incubation Procedure

Wild-type and *mdx* mice were anesthetized using isoflurane (2%, inhalation) and euthanized by cervical dislocation. Cardiomyocytes were then isolated from the ventricles of their hearts using a Langendorff setup (Hugo Sachs Elektronik, March, Germany) according to the isolation procedure described in detail in our previous work (5). In brief, the hearts were rapidly excised, and a cannula was inserted into the aorta for retrograde perfusion with Ca^2+^-free solution containing 0.17 mg/mL Liberase TH (Roche) at 37°C for 10 min. Thereafter, the ventricles were cut into pieces and incubated on a shaker at 37°C. Subsequently, the Ca^2+^ concentration was increased to 150 μM over 30 min in four steps. Pieces of digested tissue were then triturated to liberate ventricular cardiomyocytes. After a centrifugation step, the myocytes were resuspended in minimum essential medium (MEM)-α (Sigma), containing ITS media supplement (Sigma) diluted (1:100), 2 mM l-glutamine, 100 U/mL penicillin, 0.1 mg/mL streptomycin, and 17 µM blebbistatin (Sigma). Myocytes were finally plated on Matrigel (Becton Dickinson)-coated culture dishes. For incubation experiments, cells were exposed to 1 µM EMPA, 10 µM cariporide (MedChemExpress), or 10 µM cariporide in combination with 1 µM EMPA for 24 h by adding these drugs from 100 mM stock solutions in DMSO to the cell culture medium. Cardiomyocytes of the control groups were incubated with DMSO alone. DMSO concentrations were equal in all compared experimental groups. Drug-treated cardiomyocytes and DMSO-treated control cells always originated from the same cardiomyocyte isolation (identical mouse).

### *I*_Na_ Recordings

The whole cell patch clamp technique was used to record *I*_Na_ from ventricular cardiomyocytes up to 6 h after preparation (except for 24-h incubation experiments). The recordings were performed at room temperature (22 ± 1.5°C) by using an Axopatch 200B patch clamp amplifier (Axon Instruments, Union City, CA). Pipettes were formed from aluminosilicate glass (A120-77-10; Science Products, Hofheim, Germany) with a P-97 horizontal puller (Sutter Instruments, Novato, CA). They had resistances between 0.8 and 1.2 MΩ when filled with pipette solution. Data acquisition was performed with pClamp 10 software (Axon Instruments) through a 16-bit A-D/D-A interface (Digidata1440; Axon Instruments). Data were low-pass filtered with 10 kHz (−3 dB) and digitized at 35 kHz. Data analysis was performed with Clampfit 10.7 (Axon Instruments) and GraphPad Prism 8 (San Diego, CA) software. Current-voltage (*I*-*V*) relationships were fit with the function: *I* = *G*_max_·(*V* − *V*_rev_)/{1 + exp[(*V*_50_ − *V*)/*K*]}, where *I* is the current, *G*_max_ is the maximum conductance, *V* is the membrane potential, *V*_rev_ is the reversal potential, *V*_50_ is the voltage at which half-maximum activation occurred, and *K* is the slope factor. Membrane voltages were corrected for liquid junction potentials. For current density-voltage relationships, the current amplitudes at various voltages were measured. These values were then divided by the membrane capacitance to yield current densities. A holding potential of −117 mV, from which the channels were activated by depolarizing voltage steps, was chosen to guarantee full channel availability. Steady-state fast inactivation data were fit with the Boltzmann function: *I*/*I*_max_  =  1/(1 + exp[(*V* − *V*_50_)/*K*)], where *I*/*I*_max_ is the normalized current, *V* is the membrane potential, *V*_50_ is the voltage at which half-maximum inactivation occurred, and *K* is the slope factor. Recordings from ventricular cardiomyocytes were made in a bath solution that consisted of (in mM) 5 NaCl, 135 *N*-methyl-d-glucamine, 2.5 KCl, 1 CaCl_2_, 1 MgCl_2_, and 10 HEPES (pH 7.4), adjusted with HCl. The bath solution additionally contained 17 µM blebbistatin. The pipette solution contained (in mM) 5 NaCl, 110 CsF, 10 EGTA, and 10 HEPES (pH 7.3), adjusted with CsOH. A DAD-8-VC superfusion system (ALA Scientific Instruments, Westbury, NY) was used for continuous superfusion of patched cells and allowed for rapid extracellular solution changes.

### Statistical Data Analysis

Comparisons were made using a nested analysis respecting the hierarchical data structure (measurements of n cells from m animals) detailed in Sikkel et al. (20). A *P* value <0.05 was considered significantly different.

## RESULTS

**Figure 1.**
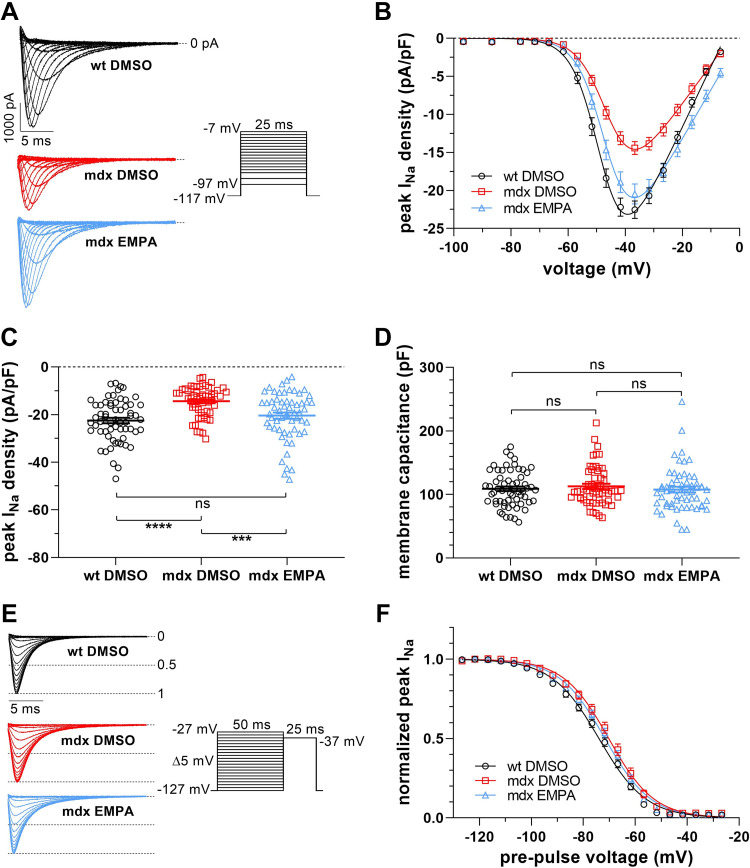
Na^+^ current (*I*_Na_) properties in ventricular cardiomyocytes derived from wild-type (WT DMSO), untreated control *mdx* (*mdx* DMSO) and EMPA-treated *mdx* (*mdx* EMPA) mice. The latter *mdx* mouse cohort had received 15 mg/kg/day EMPA via the drinking water for 4 wk; WT and control *mdx* mice had received a respective concentration of DMSO. *A*: typical original current traces of a WT, a control *mdx*, and an EMPA-treated *mdx* cardiomyocyte, elicited by the pulse protocol displayed (*inset*). *B*: from a series of such experiments [*n* = 61 cells from 4 WT hearts (black); *n* = 56 cells from 4 control *mdx* hearts (red); *n* = 58 cells from 4 *mdx* EMPA hearts (light blue)], current density-voltage relationships were derived. Data points are represented as means ± SE. Parameters for *I*_Na_ activation derived from fits of the current-voltage relationships (function described in methods) are given in Table 1. *C*: dot plot comparing the maximum peak *I*_Na_ densities of WT, control *mdx*, and EMPA-treated *mdx* cardiomyocytes at −37 mV. ****P < 0.0001, significant difference between WT and *mdx*. ***P < 0.001, significant difference between *mdx* and *mdx* EMPA. ns, not significant (*P* = 0.19). *D*: dot plot comparing membrane capacitance values for cell size estimation of WT, control *mdx*, and EMPA-treated *mdx* cardiomyocytes. ns, not significant (*P* always > 0.38). *E*: original *I*_Na_ traces of a WT, a control *mdx*, and an EMPA-treated *mdx* cardiomyocyte, elicited by a 25-ms test pulse following a series of inactivating 50-ms prepulses. The pulse protocol used to test the voltage dependence of steady-state fast inactivation is displayed (*inset*). *F*: voltage dependencies of steady-state inactivation in WT, control *mdx*, and *mdx* EMPA cardiomyocytes (*n =* 48 cells from 4 WT hearts; *n* = 31 cells from 4 control *mdx* hearts; *n* = 32 cells from 4 *mdx* EMPA hearts). Parameters for steady-state fast inactivation derived from fits with a Boltzmann function (see methods) are given in Table 1.

The effects of EMPA on *I*_Na_ of dystrophin-deficient ventricular cardiomyocytes were studied in a series of experiments. First, *I*_Na_ of myocytes derived from *mdx* mice, which had received 15 mg/kg/day EMPA via the drinking water for 4 wk, was compared with *I*_Na_ of myocytes from control *mdx* mice. Figure 1, *A* and *B*, shows that the peak *I*_Na_ density of EMPA-exposed *mdx* myocytes was markedly increased over a wide voltage range and comparable to the peak *I*_Na_ density of wild-type myocytes. At −37 mV, the voltage at which the *I*_Na_ density was maximal, a highly significant difference existed between EMPA-exposed *mdx* and control *mdx* cardiomyocytes (Fig. 1*C*). The comparison between EMPA-exposed *mdx* and wild-type myocytes revealed no significant difference (*P* = 0.19). These experiments suggested that, by long-term EMPA treatment, abnormally reduced *I*_Na_ of ventricular cardiomyocytes from dystrophin-deficient *mdx* mice can be rescued. Furthermore, Fig. 1, *E* and *F*, shows that the voltage dependence of *I*_Na_ steady-state fast inactivation was similar in wild-type, *mdx*, and EMPA-exposed *mdx* myocytes. The same was true for the voltage dependence of *I*_Na_ activation (for respective parameters of *I*_Na_ activation and inactivation see Table 1).

**Table 1.** Parameters of Na^+^ current activation and fast inactivation in ventricular cardiomyocytes

	Activation				Inactivation		
	*V*_50_, mV	*K*, mV	*V*_rev_, mV	*n*	*V*_50_, mV	*K*, mV	*n*
In vivo EMPA treatment (corresponding to Fig. 1)							
WT DMSO	−48.04 ± 0.53	4.05 ± 0.08	−4.9 ± 0.66**	61	−74.07 ± 0.99	−8.12 ± 0.16	48
*mdx* DMSO	−45.86 ± 0.64	3.99 ± 0.08	−3.12 ± 0.95*	56	−70.39 ± 1.26	−7.75 ± 0.24	31
*mdx* EMPA	−45.65 ± 0.61	4.19 ± 0.08	0.85 ± 0.97	58	−71.6 ± 1.01	−8.11 ± 0.19	32
EMPA incubation of cardiomyocytes (corresponding to Fig. 2)							
*mdx* DMSO	−46 ± 0.86	4.28 ± 0.1	−1.54 ± 1.14	36	−75.35 ± 1.91	−7.73 ± 0.33	15
*mdx* EMPA	−47.76 ± 0.79	4.01 ± 0.11	1.78 ± 1.32	43	−73.94 ± 2.02	−7.7 ± 0.18	18

Values are means ± SE; *n*, number of cells. Current-voltage (*I*-*V*) relationships were fit with the function: *I* = *G*_max_·(*V* − *V*_rev_)/{1 + exp[(*V*_50_ − *V*)/K]}, where *I* is the current, *G*_max_ is the maximum conductance, *V* is the membrane potential, *V*_rev_ is the reversal potential, *V*_50_ is the voltage at which half-maximum activation occurred, and *K* is the slope factor. **P < 0.01, significant difference between WT DMSO and *mdx* EMPA. *P < 0.05, significant difference between *mdx* DMSO and *mdx* EMPA. Steady-state fast inactivation data were fit with the Boltzmann function: *I*/*I*_max_  =  1/{1 + exp[(*V* − *V*_50_)/*K*]}, where *I*/*I*_max_ is the normalized current. EMPA, empagliflozin; WT, wild-type.

**Figure 2.**
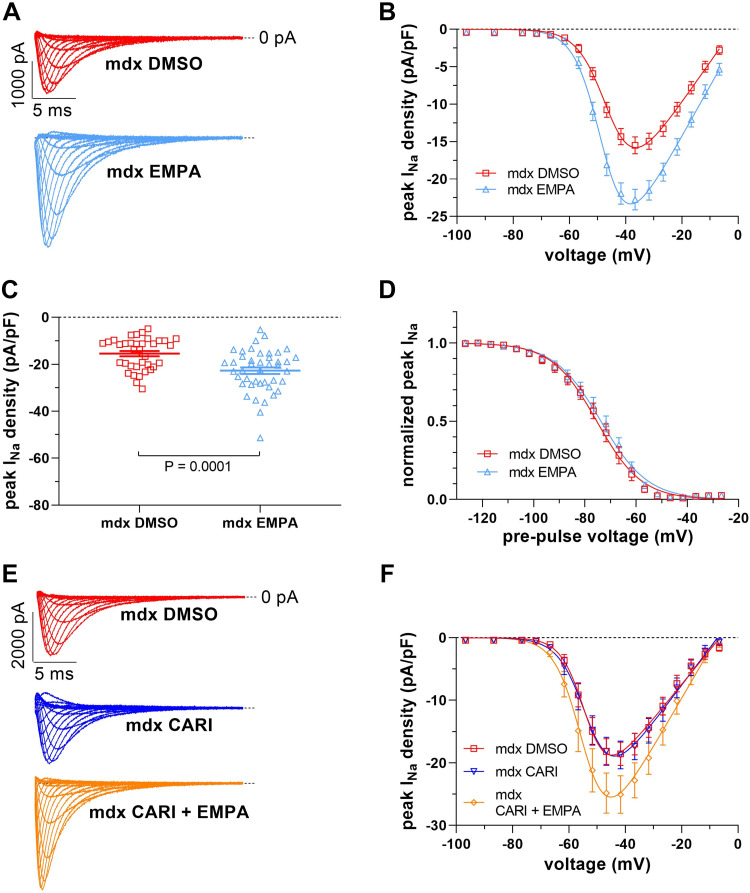
Effect of 24-h incubation with 1 µM empagliflozin (EMPA) on Na^+^ current (*I*_Na_) properties of ventricular cardiomyocytes derived from *mdx* mice. *A*: typical original current traces of a control *mdx* (*mdx* DMSO) and an EMPA-treated (*mdx* EMPA) cardiomyocyte (for pulse protocol see Fig. 1*A*). *B*: from a series of such experiments [*n* = 36 *mdx* cells (red); *n* = 43 *mdx* EMPA cells (light blue); all cells originating from 4 *mdx* hearts], current density-voltage relationships were derived. *C*: dot plot comparing the maximum peak *I*_Na_ densities of control *mdx* and EMPA-treated *mdx* cardiomyocytes at −37 mV. *P* = 0.0001, significant difference between *mdx* and *mdx* EMPA. *D*: voltage dependencies of steady-state inactivation (for pulse protocol, see Fig. 1*E*) in control *mdx* and *mdx* EMPA cardiomyocytes (*n* = 15 *mdx* cells; *n* = 18 *mdx* EMPA cells; all cells originating from 4 *mdx* hearts). *I*_Na_ activation and steady-state fast inactivation parameters are given in Table 1. *E*: original current traces of a control *mdx* (*mdx* DMSO), a cariporide-treated *mdx* (*mdx* CARI, 24-h incubation at 10 µM concentration), and a cariporide-plus EMPA-treated (*mdx* CARI + EMPA) cardiomyocyte. *F*: current density-voltage relationships derived from a series of such experiments [*n* = 16 *mdx* cells (red); *n* = 19 *mdx* CARI cells (dark blue); *n* = 15 *mdx* CARI + EMPA cells (orange); all cells originating from 2 *mdx* hearts].

We next incubated ventricular cardiomyocytes isolated from *mdx* mice with 1 µM EMPA for 24 h. Thereafter, their *I*_Na_ was measured and compared with *I*_Na_ of control *mdx* myocytes from the same preparation. Figure 2, *A–C*, shows that EMPA incubation significantly increased the peak *I*_Na_ density of *mdx* myocytes to a similar extent as in the *mdx* mouse EMPA treatment experiment described earlier. Again, the voltage dependencies of *I*_Na_ activation (Table 1) and steady-state fast inactivation (Fig. 2*D* and Table 1) were not affected by the presence of EMPA. One of the potential targets of EMPA in the heart is the Na^+^-H^+^ exchanger 1 (NHE-1), which is known to be inhibited by the drug (21–23). To test the potential involvement of NHE-1 in the observed EMPA effect on *I*_Na_ of *mdx* ventricular cardiomyocytes, we used the selective NHE-1 inhibitor cariporide. Figure 2, *E* and *F*, shows that incubation of *mdx* myocytes with 10 µM cariporide (23, 24) for 24 h had no effect on the peak *I*_Na_ density. Furthermore, the presence of cariporide during EMPA incubation did not affect EMPA’s enhancing effect on the peak *I*_Na_ density. These experiments suggested that EMPA likely acts via another mechanism than NHE-1 inhibition.

**Figure 3.**
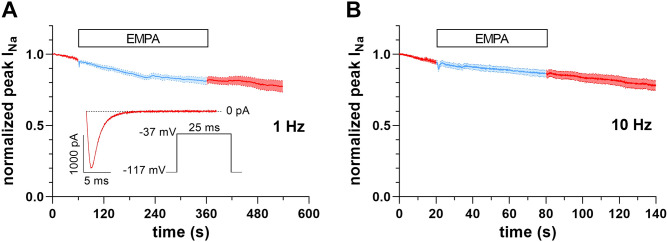
Lack of acute empagliflozin (EMPA) effects on peak Na^+^ current (*I*_Na_) of *mdx* ventricular cardiomyocytes. *A*: peak *I*_Na_ before, during superfusion with bath solution containing 1 µM EMPA (light blue), and after washout over time (*n* = 13 cells from 2 *mdx* hearts). Data are expressed as means ± SE. *Inset*: pulse protocol, applied at 1-Hz frequency and elicited *I*_Na_ at the very beginning of the experiment. *B*: equivalent experiment as described in *A* at 10-Hz frequency (*n* = 15 cells from 2 *mdx* hearts).

In a final set of experiments, we tested the acute effects of EMPA on peak *I*_Na_ of *mdx* ventricular cardiomyocytes. Figure 3, *A* and *B*, shows that superfusion of *mdx* myocytes with bath solution containing 1 µM EMPA did not affect peak *I*_Na_. Acute application of 10 µM EMPA also did not impact peak *I*_Na_ (data not shown). These experiments suggested that EMPA does not acutely modulate peak *I*_Na_ of dystrophin-deficient ventricular cardiomyocytes.

## DISCUSSION

Here, we report that ventricular cardiomyocytes derived from dystrophin-deficient *mdx* mice, which had received 15 mg/kg/day EMPA via the drinking water for 4 wk, showed peak *I*_Na_ densities comparable with myocytes from wild-type mice. Furthermore, 24-h incubation of isolated *mdx* ventricular cardiomyocytes with 1 µM EMPA significantly enhanced their *I*_Na_. EMPA treatment studies with mice are typically conducted in a dose range between 10 and 35 mg/kg body wt/day (16–19). These doses were shown to lead to therapeutic plasma concentrations (19), and 1 µM EMPA is considered a therapeutically relevant concentration (11, 12, 14). Consequently, our findings suggest that long-term treatment with therapeutic concentrations of EMPA rescues peak *I*_Na_ loss in dystrophin-deficient ventricular cardiomyocytes.

### Potential Mechanism(s) of Action

In our hands, EMPA did not exert acute effects on peak *I*_Na_ of dystrophin-deficient ventricular cardiomyocytes (Fig. 3). This agrees with Philippaert et al. (11), who reported that acutely applied EMPA had only little effect on peak *I*_Na_ of cardiomyocytes from mice with HF. Interestingly, in contrast to peak *I*_Na_, late *I*_Na_ was significantly inhibited by acute EMPA application, and these authors proposed a direct interaction of the drug with the Na_v_1.5 Na^+^ channel. Other authors, however, did not observe acute EMPA effects on late *I*_Na_ of HF cardiomyocytes, but reported that the drug inhibited late *I*_Na_ only after a prolonged incubation period (12). Rather than a direct interaction of EMPA with Na_v_1.5, this suggested an indirect mechanism, possibly via inhibition of Ca^2+^/calmodulin-dependent protein kinase-II (CaMKII) by the drug (12, 13). Future studies should clarify this issue.

Increased peak *I*_Na_ of dystrophin-deficient ventricular cardiomyocytes under sustained presence of EMPA without considerably altered channel gating (Figs. 1 and 2; Table 1) suggests drug-induced upregulation of Na_v_1.5 channel protein expression. Enhancement of Na_v_1.5 expression by EMPA in the dystrophic heart accords with significantly increased *Scn5a* gene transcript levels observed in cardiac samples from SGLT2 inhibitor-treated HF mouse models (25, 26). With regard to EMPA’s potential mode(s) of action, we can only speculate. Since SGLT2 is not or hardly expressed in cardiac tissue (27–29), EMPA must exert its effects on Na_v_1.5 expression in dystrophin-deficient cardiomyocytes via another mechanism than SGLT2 inhibition. A potential alternative target is NHE-1, which is known to be inhibited by EMPA (21–23). Here, we show that incubation with cariporide, a selective NHE-1 inhibitor, had no effect on peak *I*_Na_ of dystrophin-deficient cardiomyocytes, and that the presence of cariporide during EMPA incubation did not affect EMPA’s impact on peak *I*_Na_ (Fig. 2, *E* and *F*). This suggests that inhibition of NHE-1 by EMPA can be excluded as the underlying mechanism under our experimental conditions. Another conceivable mode of action of EMPA is modulation of intracellular Ca^2+^ handling with secondary impact on Na^+^ channel expression. Several research laboratories previously reported that EMPA and other SGLT2 inhibitors significantly improve impaired Ca^2+^ handling properties in HF cardiomyocytes (23, 27, 30–32). Among these improvements, a reduction in diastolic (resting) Ca^2+^ in the presence of a SGLT2 inhibitor was observed (31). A decrease in resting Ca^2+^ was also found in isolated healthy murine cardiomyocytes after an EMPA exposure for 24 h (30). Diminished resting Ca^2+^ levels are known to induce an upregulation of Na^+^ channel expression in myocytes (33, 34). We therefore speculate that EMPA-induced upregulation of Na_v_1.5 expression in dystrophin-deficient ventricular cardiomyocytes may result from reduced resting Ca^2+^ levels in the presence of the drug. EMPA’s actual mode of action remains to be elucidated.

### Study Limitations and Future Directions

Although we assume that the enhancement of peak *I*_Na_ of dystrophin-deficient ventricular cardiomyocytes due to EMPA treatment results from increased Na_v_1.5 channel expression, we have not confirmed this experimentally. Alternative explanations would be drug-induced upregulation of noncardiac Na^+^ channel isoforms, alterations in Na^+^ channel localization, or enhancement of channel conductance. Furthermore, we encourage researchers to test a potential involvement of CaMKII, which reduces peak *I*_Na_ of cardiomyocytes (35). EMPA inhibits CaMKII (12, 13) and may thereby have an enhancing effect on peak *I*_Na_. In the present study, we have not tested EMPA effects on peak *I*_Na_ of control ventricular cardiomyocytes from wild-type mice. Significantly increased peak *I*_Na_ of cardiomyocytes derived from human-induced pluripotent stem cells after EMPA incubation (14), however, suggests that the drug’s effect does not only occur in case of dystrophin deficiency. The effect of EMPA on late *I*_Na_ in dystrophin-deficient ventricular cardiomyocytes was also not studied here, because there is no evidence in the literature for the occurrence of enhanced late *I*_Na_ in dystrophin-deficient versus wild-type myocytes. Furthermore, EMPA effects on peak *I*_Na_ of dystrophin-deficient cardiac Purkinje fibers (7, 8) should be tested, because this cell type is a major determinant of ventricular conduction velocity. Finally, we encourage a comparison of the arrhythmia incidence in EMPA-treated versus untreated *mdx* mice.

### Clinical Relevance

SGLT2 inhibitors have recently been approved for the treatment of patients with HF but not diabetes. Among various potential beneficial effects for the diseased heart, these drugs are antiarrhythmic, whereby inhibition of late *I*_Na_ of HF cardiomyocytes is considered a relevant mechanism (11–13). To the best of our knowledge, the effectiveness of SGLT2 inhibitors in DMD cardiomyopathy is unexplored. Significantly reduced peak *I*_Na_, a characteristic feature of dystrophin-deficient ventricular cardiomyocytes (4–8), represents a relevant source of cardiac arrhythmias. Accordingly, *Scn5a*^+/−^ mice (animal model for the Brugada syndrome) (36, 37), which have a similarly reduced cardiomyocyte peak *I*_Na_ as *mdx* mice (4–6), show ventricular arrhythmias and conduction disorders (36). Here, we report that EMPA treatment of *mdx* mice completely rescues peak *I*_Na_ loss in dystrophin-deficient ventricular cardiomyocytes. Thereby, we have corrected the source of impaired ventricular conduction and concomitant arrhythmias in the dystrophic heart. We speculate that EMPA treatment may improve ventricular conduction and prevent arrhythmias in human patients with DMD. EMPA may also be useful for the treatment of other arrhythmic disorders triggered by Na^+^ channel loss of function.

Collectively, our study implies that EMPA treatment can rescue abnormally reduced peak *I*_Na_ of dystrophin-deficient ventricular cardiomyocytes. Further studies are needed to explore whether this effect is EMPA specific or common to SGLT2 inhibitors in general, and whether EMPA administration can diminish arrhythmia vulnerability in patients with DMD.

## DATA AVAILABILITY

The data underlying this article will be shared on reasonable request to the corresponding author.

## GRANTS

This work was supported by Austrian Science Fund (FWF) Grants P35542-B and P35878-B (to K. Hilber).

## DISCLOSURES

No conflicts of interest, financial or otherwise, are declared by the authors.

## AUTHOR CONTRIBUTIONS

J.S., A.K., and K.H. conceived and designed research; J.S., J.M., E.L., and B.H. performed experiments; J.S., J.M., B.H., and C.D. analyzed data; J.S., H.T., H.K., B.K.P., A.K., X.K., and K.H. interpreted results of experiments; J.S., E.L., and C.D. prepared figures; J.S., X.K., and K.H. drafted manuscript; H.T., H.K., B.K.P., A.K. X.K., and K.H. edited and revised manuscript; J.S., J.M., E.L., B.H., H.T., H.K., C.D., B.K.P., A.K., X.K., and K.H. approved final version of manuscript.
